# Editorial: Inorganic particles and fibres: integrating minero-chemistry and hazard assessment for eco-exposome development

**DOI:** 10.3389/fchem.2023.1233846

**Published:** 2023-07-03

**Authors:** Jasmine Rita Petriglieri, Cristina Pavan, Andrij Holian, Francesco Turci

**Affiliations:** ^1^ “G Scansetti” Interdepartmental Centre for Studies on Asbestos and Other Toxic Particulates, University of Torino, Turin, Italy; ^2^ Department of Earth Sciences, University of Torino, Turin, Italy; ^3^ Louvain Centre for Toxicology and Applied Pharmacology, Université Catholique de Louvain, Bruxelles, Belgium; ^4^ Center for Environmental Health Science, Department of Biomedical and Pharmaceutical Sciences, University of Montana, Missoula, MT, United States; ^5^ Department of Chemistry, University of Torino, Turin, Italy

**Keywords:** inorganic particles and fibres, hazard assessment, toxicity mechanisms, mineral eco-exposome, asbestos, silica dust, hazard and risk, inorganic pollutants

Exposure to mineral particles and fibres is the leading cause of respiratory diseases and malignancies, mainly in occupational settings. Exposure to respirable crystalline silica (RCS), mainly in the form of *α*-quartz, is related to the risk of developing silicosis, lung cancer, and autoimmune diseases. Silicosis still represents a major occupational Research Topic worldwide. The enforcement of strict safety standards and worker protection measures have lowered the level of occupational exposure and decreased the incidence of related lung diseases, in some countries. However, this success is mainly limited to high-income countries and new occupational and/or environmental hazard may arise from new materials or emerging pollutants (EP), as well-illustrated by the cases of accelerated silicosis from artificial stone (AS) dusts or the risk posed by natural occurrences of asbestos and asbestos-like minerals (NOA) (Leso et al., 2019; Campopiano et al., 2020; Petriglieri et al., 2021; Petriglieri et al., 2023). In fact, exposure to asbestos, silica, and other mineral dusts is still responsible for 25% of all occupational lung diseases (Cullinan et al., 2017; Thives et al., 2022). Diseases associated with the exposure to new material dusts and non-conventional exposure scenarios require new strategies for risk assessment, monitoring, and mitigation. Under such circumstances, the risk is often posed by exposure to mixtures of inorganic particles, which overall toxicity is largely unknown. In the realm of mineral fibres, rock surfaces may be exposed to weathering and erosion and increase the environmental presence of potentially hazardous fibres (Lee et al., 2008; Koumantakis et al., 2009; Freemantle et al., 2022; Malinconico et al., 2022; Walter et al., 2022). Fibrous minerals released during weathering processes may share with asbestos minerals some of their key physico-chemical and toxicological characteristics and may ultimately pose a risk to human health, if inhaled (Cardile et al., 2006; Harper, 2008; NIOSH, 2011; Boffetta et al., 2018; Garabrant and Pastula, 2018; Gazzano et al., 2023). The pathogenic potential of an inhalable poorly soluble mineral particle depends on some key minero-chemical aspects, specifically: i) the particle size and shape, which define the aerodynamic diameter and regulate the access to alveolar space and translocation to secondary target organs (Geiser and Kreyling, 2010); ii) the particle crystal structure and chemistry, which determines the leaching processes within the lung and consequently define the bio-solubility, the surface reconstruction, and surface reactivity upon particle inhalation (Straif et al., 2009; Donaldson and Seaton, 2012; IARC, 2012). The combination of these aspects determines the residence time of the particle within the lung, which is directly related with the long-term health effect of a xenobiotic particle.

To design an integrated occupational and environmental strategy for hazard assessment and control, the toxicity paradigms for airborne toxic particles have to be extended to the whole realm of inorganic dusts also in non-conventional exposure scenarios. To tackle this challenge, we must advance our knowledge on the complex interplay established by inorganic particles and biological environments. In parallel, new exposure scenarios of well-known or still poorly-described inorganic dusts, e.g., quartz (Pavan et al., 2016), asbestos (Gualtieri, 2023), or celestial dusts (Corazzari et al., 2021), should be investigated in terms of adequate risk assessment, monitoring, and mitigation strategies.

To cover promising, recent, and novel research trends in all the above mentioned aspects of toxic or potentially toxic inorganic particles and fibres, we challenged the scientific community to advance the development of mineral particle eco-exposome, which represents the Frontier for assessing the effects of multiple exposures to chemicals. This challenging task translates, for inorganic particles and fibres, in the design of integrated investigation strategies that will bridge minero-chemical properties to hazard assessment.

The contributions collected spanned from the definition of the geological occurrence of a well-known carcinogenic fibre to the description of the molecular interactions taking place at the bio-inorganic interface of silica and silicates.

The work by Patel et al. reviewed almost 200 references to define the new state-of-the-art in the geolocalization of the occurrence of erionite worldwide. Erionite is a fibrous zeolite which has been recognized as the causal agent to lung cancer and malignant mesothelioma. The extensive analysis of the literature provides more than 100 global locations where erionite has been reported. In addition to reporting the geological setting of host rocks and the paragenetic sequence of erionite formation, the paper includes a description of the fiber morphological and mineralogical properties and an overview of the techniques to identify and characterize the mineral. With an analytical perspective, the work by Avataneo et al. dealt with the emerging concern of the presence of asbestos fibres in water. To compensate for the limited attention that has been dedicated to waterborne asbestos, this experimental work mimicked a Scanning Electron Microscopy analysis of waterborne chrysotile from stream water and tested the reliability of the method with a multi-instrument, multi-operator double check of samples. Good practices to perform reliable analyses on surface water samples containing asbestos were proposed. To investigate the overlooked presence of emerging contaminants such as naturally occurring fibre-like particles (FLP), Talbot et al. performed a re-examination of several particulate matter filters from three sites across Auckland (NZ) that were collected during a 20-year long air monitoring campaign. The analysis confirmed the occasional presence of erionite in the re-analysed filters. A micro-dispenser method to prepare depositions of asbestos fibres for toxicological tests *in vitro* is reported by Della Ventura et al. This novel approach deposit micro-sized droplets from a suspension of fibres and allows control of some key parameters including the deposition area, the deposition time, and the uniformity and volume of the deposited liquid. Advances on the description of the molecular interactions taking place between inorganic particles and model membranes are reported in the collective Research Topic. Moving from the interaction paradigm proved to occur between cellular membranes and a specific population of weakly interacting silanols exposed at quartz surfaces, the nearly free silanols (Pavan et al., 2020; Friesen et al., 2022; Pavan et al., 2022; Pavan et al., this issue), Pavan et al. expanded their interaction model to the other crystalline silica polymorphs and paved the way to a comprehensive understanding of the structure-activity relationship between nearly free silanols and membranolytic activity of crystalline silica. With a complementary approach focussed on membrane chemistry, Sydor et al. showed that an increase in the cholesterol content of membranes can attenuate the silica-induced membrane alterations, ultimately suggesting that the selective manipulation of lysosomal cholesterol may be a way of mitigating silica-induced lysosomal disruption and preventing chronic inflammatory disease progression.

Overall, this Research Topic has put together some novel and relevant research that can pave the way for a more integrated comprehension of how inorganic chemistry, analytical approaches and toxicological investigations can be integrated to develop an eco-exposome approach to particle and fibre hazard assessment.
